# Nanoconfined Water in Pillared Zeolites Probed by ^1^H Nuclear Magnetic Resonance

**DOI:** 10.3390/ijms242115898

**Published:** 2023-11-02

**Authors:** Marina G. Shelyapina, Denis Y. Nefedov, Anastasiia O. Antonenko, Gleb A. Valkovskiy, Rosario I. Yocupicio-Gaxiola, Vitalii Petranovskii

**Affiliations:** 1 Faculty of Physics, Saint Petersburg State University, 7/9 Universitetskaya Nab., Saint Petersburg 199034, Russia; iverson89@yandex.ru (D.Y.N.); a.antonenko@spbu.ru (A.O.A.); g.valkovsky@spbu.ru (G.A.V.); 2 Tecnológico Nacional de México, Instituto Tecnológico Superior de Guasave, Carretera a La Brecha Sin Número, Ejido Burrioncito, Guasave 81149, Sinaloa, Mexico; rosario.yg@guasave.tecnm.mx; 3 Center for Nanoscience and Nanotechnology, National Autonomous University of Mexico (CNyN, UNAM), Ensenada 22860, Baja California, Mexico; vitalii@ens.cnyn.unam.mx

**Keywords:** hierarchical zeolites, mordenite, ZSM-5, water dynamics, proton NMR

## Abstract

Here, we report the results of our ^1^H nuclear magnetic resonance study of the dynamics of water molecules confined in zeolites (mordenite and ZSM-5 structures) with hierarchical porosity (micropores in zeolite lamella and mesopores formed by amorphous SiO_2_ in the inter-lamellar space). ^1^H nuclear magnetic resonance (NMR) spectra show that water experiences complex behavior within the temperature range from 173 to 298 K. The temperature dependence of ^1^H spin-lattice relaxation evidences the presence of three processes with different activation energies: freezing (about 30 kJ/mol), fast rotation (about 10 kJ/mol), and translational motion of water molecules (23.6 and 26.0 kJ/mol for pillared mordenite and ZSM-5, respectively). For translational motion, the activation energy is markedly lower than for water in mesoporous silica or zeolites with similar mesopore size but with disordered secondary porosity. This indicates that the process of water diffusion in zeolites with hierarchical porosity is governed not only by the presence of mesopores, but also by the mutual arrangement of meso- and micropores. The translational motion of water molecules is determined mainly by zeolite micropores.

## 1. Introduction

Porous solids are widely used in chemical, energy and environmental processes where the controlled transport of molecules through pores plays a crucial role. They are intensively used as catalysts and catalytic supports. According to IUPAC, these porous materials can be classified into microporous (*d* < 2 nm), mesoporous (2 < *d* < 50 nm) and macroporous (*d* > 50 nm) depending on their pore sizes *d*. The relationship between the pore diameter and the size of reacting molecules leads to the need to create hierarchical structures, in which transport diffusion pores are present. 

Confinement effects in porous media, which are experienced by adsorbed molecules, lead to significant deviations from bulk diffusion, which allow adjusting conditions of catalytic reactions. Significant progress in the field of synthesis makes it possible to realize in practice the preparation of materials in a wide range of sizes, shapes and topologies of pores [1].

Layered mesoporous zeolites containing hierarchically structured micro-, meso- and/or macropores are of increasing interest for use as catalysts in reactions involving bulk molecules because they facilitate their transport over macroscopic distances. Since these materials contain several different types of porosity and thus each have qualitatively different surfaces for contacting molecules, it is important to study the details of these interactions. Water molecules are a convenient and natural probe to study in detail the contact between polar molecules and porous materials. The obtained knowledge about the structure of complexes and their dynamics with participation of all structural elements of porous materials is essential for the successful development of new catalysts and adsorbents.

At the same time, the behavior of water confined in all kinds of porous materials and, in particular, in zeolites, is of independent interest because it is an important key to understanding various phenomena occurring in natural environments, including mineralogy, geophysics, and ecology. Physicochemical processes such as adsorption, desorption, diffusion, and molecular formation reactions on the surface of cosmic dust particles play a central role in the physics and chemistry of the space environment and are responsible for the synthesis of a significant fraction of key astronomical molecules [2].

Water has long been investigated as an essential solvent, including studies of phase transitions in water under a wide variety of conditions, both for bulk water and its nanodispersed states. The influence of the size of water droplets and/or layers on the temperature of phase transitions has been studied by inelastic neutron scattering [3,4], positron annihilation [5], dielectric relaxation spectroscopy [6], and nuclear magnetic resonance (NMR) methods [7,8,9,10], among many others. Water itself, even in the bulk state, exhibits many different coexisting structures [11]. At the nanoscale, this diversity increases even further [12,13,14,15]. An interesting concept about the possibility of fine-tuning the properties of water as a solvent is proposed in Ref. [16]. The authors suggest that confining water in hydrophobic nanopores could be a way to modulate the solvent properties of water, allowing use of water as a tunable solvent (WaTuSo). 

The dynamics of water on a mineral substrate play an important role in interfacial processes. This is because the structure and dynamics of interfacial water are different from those of bulk water due to changes in the interactions between surface water molecules, and interactions between surface water and substrate [4]. Due to the high polarity of water molecules and their ability to form a branched network of hydrogen bonds among themselves, bulk water, both in liquid and solid states, exhibits a very high internal cohesiveness [17]. 

In confined geometries, water molecules can interact both with surrounding surfaces and with each other. Competition between these interactions can lead to new elements of water structure. Mesoporous silica [8,18,19,20,21] or zeolites [7,19] are clear examples of the nanoconfinement effect on the structure and behavior of water.

Both the structure and dynamics of water can be probed by proton NMR. The large inner surface area and significant internal volume of these mesoporous materials provide a considerable amount of adsorbed water, which allows a detailed analysis of water behavior at the molecular scale by NMR techniques. As soon as the ^1^H chemical shift is sensitive to the structure and binding of individual water molecules, it can be used to probe the local environment [22]. 

In turn, the main issues responsible for the NMR relaxation in water are fluctuating strengths of ^1^H–^1^H dipole coupling. As these interactions depend on the relative positions of the interacting nuclear spins, they are altered by motional processes. This makes it possible to use ^1^H relaxation to study the dynamics of water. Temperature dependences of proton spin-lattice relaxation rates, *R*_1_, besides providing information concerning parameters of water molecule motion (activation energy and correlation time values), reflect the phase undergone by confined water with temperature. According to several studies, the proton spin-lattice relaxation rate and the low-temperature transition point of confined water in mesoporous materials are linearly correlated with the inverse pore radius in these materials [18,23,24].

In our previous work, we reported on the synthesis of pillared zeolites with different topology, in which zeolite lamellae with thickness of about 0.75 nm (mordenite) and 2.0 nm (ZSM-5), were separated by amorphous SiO_2_ pillars forming mesoporosity [25] that can be occupied by water molecules. A detailed study of the local structure of their zeolite framework and its evolution at different preparation steps probed by multinuclear NMR was reported in Ref. [26]. 

Herein, we report the results of our ^1^H NMR study of the dynamics of water molecules confined in the total volume (in the zeolite micropores and interlamellar space) of 2D zeolites pillared by silicon dioxide with mordenite (MOR-P) and ZSM-5 (MFI-P) structures.

**Figure 1:**
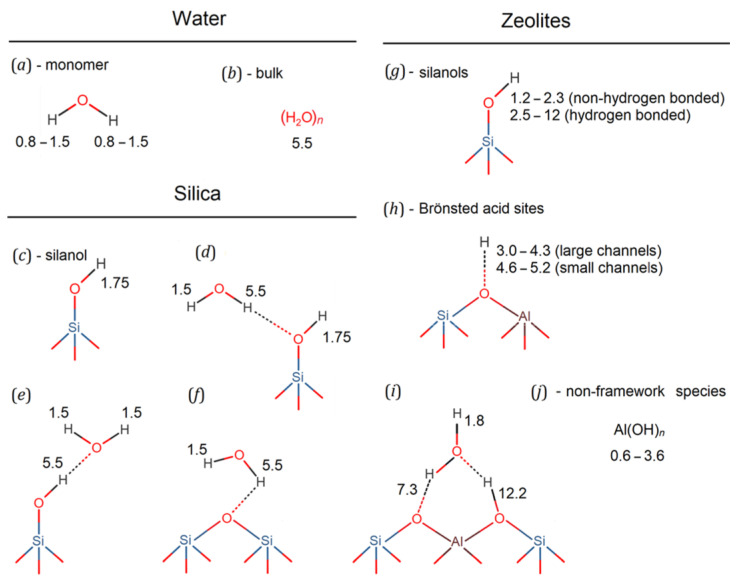
^1^H chemical shift in ppm (relative to TMS) for possible –OH groups in water (**a**,**b**), water–silica systems (**c**–**f**) [8], and zeolites (**g**–**j**) [27,28,29,30,31,32].

However, before discussing the experimental results obtained, let us provide a brief overview of the most commonly observed ^1^H chemical shifts in zeolite materials, and their relation to water structures that may occur within mesopores. Figure 1 summarizes the structural schematics of the various OH-containing species, with the corresponding proton chemical shifts indicated. 

The protons of an isolated water molecule have a chemical shift within the range from 0.8 to 1.5 ppm, Figure 1a. However, in bulk water, the molecules form a branched network of hydrogen bonds, resulting in a significantly larger chemical shift, typically around 5.5 ppm, Figure 1b. In mesoporous silica, protons of surface silanol groups are observed at 1.75 ppm, Figure 1c. According to the data reported in Ref. [8], different scenarios are possible for each of the water molecules to establish a network of hydrogen bonds. In this case, any of the water molecules can form a network of hydrogen bonds with the silica surface, or locally with each other, or to be embedded in an array of bulk water; depending on the local environment, each of the protons is characterized by an individual chemical shift, Figure 1d–f. 

Unlike silica, in zeolites, the partial substitution of Si atoms by Al atoms leads to a greater diversity of OH-groups: various silanols (silanols without Al as a nearest neighbor, silanols with Al as a nearest neighbor, linked via a non-protonated or protonated O-atom, Al-substituted silanol), Brønsted acid sites, non-framework Al(OH)_n_ species, etc. (we continue to refer to the surface OH groups as “silanol” groups, regardless of whether they are on a purely Si silica surface site, or coordinated to an Al-containing tetrahedron of the zeolite structure). A partial substitution of Si by Al distorts the structural tetrahedron to which the silanol OH-group is coordinated, altering the electron density distribution around it. For example, as shown by calculations within density functional theory (DFT) on the example of the MFI zeolite structure, the Al atom, to which the silanol is coordinated, distorts the local environment in such a way as to allow the formation of strong H bonds with vicinal silanols, weakening the Al-OH interaction [29]. The magnitude of the ^1^H chemical shift for zeolitic silanols varies between 1.2 and 2.3 ppm for silanols without hydrogen bonds and between 2.5 and 12 ppm for silanols with hydrogen bonds, see Figure 1g [28]. 

Brønsted acid sites (BASs), which in zeolites are usually tetra-coordinated aluminum sites with bridging OH groups, are of great importance for catalysis. In dehydrated zeolites, the chemical shifts corresponding to BASs are 3.0–4.3 ppm for large channels, and 4.6–5.2 ppm for small channels, see Figure 1h [30]. In hydrated zeolites, according to quantum-chemical calculations, the proton chemical shift for hydrogen-bonded OH can be as high as 12.2 ppm, see Figure 1i [31].

The interaction of water with silanols also plays an important role in the dealumination process. According to recent DFT studies, adsorption of water on Al in antiposition to the BAS leads to the formation of pentahedral or distorted tetrahedral Al atom with sequential hydrolysis of the Al-O bond up to the displacement of framework Al to a non-framework position [32]. The chemical shift of protons in non-framework Al species, such as Al(OH)*_n_*, is typically between 1.2 and 3.6 ppm (see Figure 1j) [28], but for example, in zeolite H-BEA, the signal at 0.6 ppm has been attributed to non-acidic non-framework AlOH groups [33]. 

In addition, there are siloxy defects (SiO–), which can appear upon synthesis to compensate for the positive charge of organic structure-directing agents [34]. In these defects, the oxygen valence is close to one instead of two for Si–O–Si bridges; thus, they are stabilized by a SiO–…HOSi hydrogen bond with a length of about 1.68 Å, corresponding to a proton chemical shift of 10.2 ppm [31]. Finally, the 9–15 ppm peak, if any, is often attributed to surface-stabilized H_3_O^+^ ions [35,36,37,38]. 

As can be seen from the above and briefly summarized in Figure 1, there is a large variety of different OH-containing species in zeolites, especially in hydrated ones, and quite often their ^1^H NMR signals overlap. Moreover, dynamic effects can lead to an exchange between different chemical shifts, resulting in partial or even complete averaging of the positions of the ^1^H NMR lines, making their assignment difficult. Nevertheless, taking into account that (i) in fully hydrated zeolites, the water signal dominates, masking the majority of the surface hydroxyl groups; (ii) the thermal behavior of the different species is different (e.g., a hydroxyl group with a stronger acid strength shows a larger change in chemical shift with temperature [39]), it is possible to attribute individual lines to different protons, and even follow up the structural changes in nanoconfined water that take place upon cooling.

## 2. Results

### 2.1. Textural Analysis

**Figure 2:**
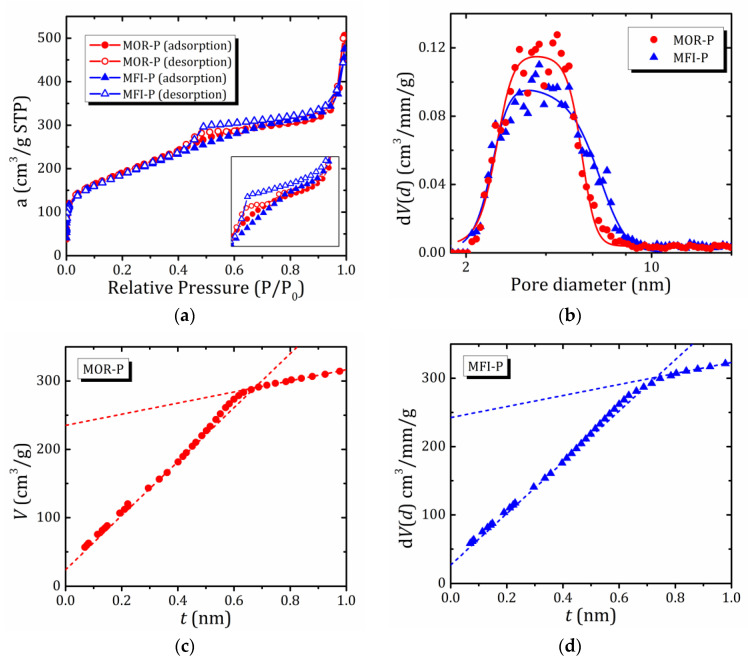
(**a**)—N_2_ adsorption/desorption isotherms of the studied pillared zeolites; (**b**)—pore size (*d*) distribution for the studied pillared zeolites obtained by NLDFT (solid lines represent a guide to the eye); (**c**,**d**)—*t*-plots for the studied pillared MOR-P and MFI-P zeolites, respectively.

The N_2_ physisorption isotherms of the samples under study are shown in Figure 2a. They can be attributed to type IV(a) isotherms according to the IUPAC classification [33,34,35], since they are characterized by the presence of hysteresis, indicating capillary condensation in mesopores. At the same time, the steep uptake at very low *P*/*P*_0_ indicates the enhancement of adsorbent–adsorptive interactions in micropores (filling of micropores in the pores of molecular dimension, i.e., of a width < ~1 nm) [33]. In other words, the observed isotherms indicate the simultaneous presence of both meso- and micropores. Moreover, the samples show a significant increase in nitrogen adsorption at high *P*/*P*_0_ values. This may be evidence of the presence of macropores and/or aggregates of disoriented small particles.

The shape of the hysteresis loops is such that it can be considered as a combination of two loops: of H2(a) and H4 types according to the IUPAC classification [33,34]. It is generally accepted that the H2 type indicates mesoporous structures with a pore shape distribution. More specifically, it refers to complex pore systems such as pore networks with ink-bottle-shaped pores, which implies that larger mesopores can only be accessed through narrow necks [34,36]. In the case of small neck widths (<5–6 nm), pore evaporation/desorption is delayed, and the wide pore body remains filled until the neck evaporates at low pressure. In particular, one can assume that desorption occurs via cavitation, or spontaneous bubble nucleation in the pore. The H4 type loop is characteristic of materials containing micro- and meso-/macropores [34]; in this case, the adsorption branch possesses a pronounced uptake at very low *P*/*P*_0_, associated with the filling of micropores, as discussed above [33]. H4 loops are often found in aggregated zeolite crystals and some mesoporous zeolites. A sharp decline in the desorption branch in the region of 0.45–0.50 *P*/*P*_0_ (in the case of nitrogen at 77 K) is typical for many compounds with a layered structure, e.g., possessing plate-like particles [33].

Generally, the sorption curves for the two samples are very similar, while the difference in the shapes of the hysteresis loops indicates the different shapes of the pores in the samples and their different size and shape distribution. Figure 2b shows the pore size distribution obtained by the DFT method using the QuadraWin program with the most appropriate of the possible models (NLDFT-N2—silica adsorption branch kernel at 77 K based on a cylindrical pore model), recommended in the case of H2 and H3 hysteresis loops and allowing for the presence of “pore network” effects such as cavitation.

The specific surface area of the samples was determined by a widely used BET method (Table 1). However, for many hierarchically structured micro-mesoporous materials, the BET area represents only an apparent surface area or “fingerprint” of a particular adsorbent [34]. Nevertheless, the BET method can be applied in the case of a IV(a) type isotherm. The results of pore sizes determination by the BJH (Barrett, Joyner and Halenda) method are also given, but only for illustrative/methodological purposes: based on a macroscopic model (Kelvin equation), BJH underestimates the mesopore size for small mesopores.

The pore size distribution shows peaks for both samples (Figure 2b); for the MOR-P sample, the peak is centered at 4.0 nm, while for MFI-P the maximum is broader and asymmetric with the center of gravity shifted towards large sizes, possibly indicating a bimodal distribution. The results obtained are in qualitative agreement with the distances between the centers of zeolite lamellae determined from small-angle X-ray scattering (SAXS) data (4.0 and 5.2 nm, respectively) [26]. In other words, MFI-P is characterized by larger mesopores with a broader size distribution.

Figure 2c,d show *t*-plots obtained by converting the relative pressure into the effective thickness (*t*) of the adsorbed layer according to the formulas provided in Refs. [38,39]. Linear approximation of the *t*-plots in two ranges of 0.32–0.42 nm and over 0.646 nm makes it possible to obtain the sum of mesopore and external surface areas *S*_meso+ext_ and micropore volume *V*_micro_, as well as external surface area *S*_ext_ and total pore volume *V*_tot_. The results obtained are summarized in Table 1. It should be noted that the small contribution of micropores in our case (small fraction of the volume and specific surface area attributable to micropores, namely ~10%) allows us to use the *t*-plot method without additional correction [39]. It is true that some results of *t*-plot analysis have only relative significance, e.g., *V*_tot_ cannot be determined in the presence of macropores, and *V*_micro_ can only serve as an order of magnitude estimate, taking into account that the *t*-plot usually underestimates *V*_micro_ [40].

In Table 1, the most credible results are highlighted in bold. Results containing a systematic error due to the inapplicability of a certain method for the corresponding analysis are italicized, so these results are provided for illustrative or methodological purposes only, for example, the BJH method, as it was commented on above. The total pore volume *V*_tot_ cannot be determined in the presence of macropores (a significant increase in nitrogen adsorption is observed at high values of *P/P*_0_).

Thus, both samples are characterized by a similar texture, although there is some variance in the shape of hysteresis loops, which indicates a slight difference in the distribution of pore size and shape. The mesopores are smaller in MOR-P, but at the same time, the specific surface area attributable to mesopores is somewhat larger. In addition, the size distribution of mesopores for MOR-P is narrower compared to MFI-P, indicating a more uniform structure of mesopores in this pillared zeolite.

### 2.2. Thermal Analysis

**Figure 3:**
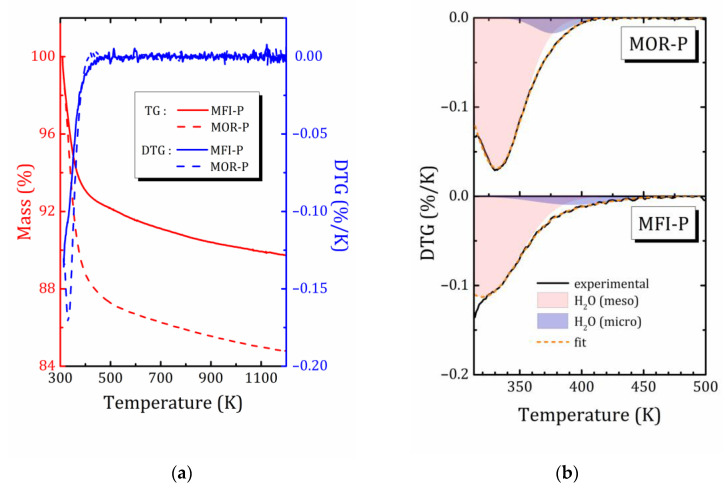
TG/DTG profiles of the MOR-P and MFI-P samples (**a**) and decomposition of DTG curves on two Gaussian lines (**b**).

According to the TG and differential TG (DTG) analysis, presented in Figure 3, the mass loss in the studied pillared zeolites upon heating occurs in several steps that can be associated to the release of water from mesopores (major water loss below 373 K), then presumably from micropores or from stronger centers, on which water is physically adsorbed (a small DTG peak around 373–423 K) [41,42], and finally to dehydroxylation (a monotonous linear mass loss in TG, not apparent in DTG) [42,43].

The mordenite sample is characterized by both higher water content and higher temperature of water release from mesopores (331 and 319 K for the pillared samples of mordenite and ZSM-5, respectively, according to DTG data, Figure 3b). Moreover, DTG clearly shows that MFI-P contains a considerable amount of weakly bound physically adsorbed surface water, which it loses at temperatures below 313 K. Altogether, this indicates differences in the texture of these two materials.

It should be noted that the TG/DTG curves of pillared zeolites MOR-P and MFI-P have features very similar to those of mesoporous silica gel containing isolated single, geminal, and vicinal (or bridged) silanol groups, for which dehydroxylation consists of the subsequent disappearance of the first vicinal silanols with an increase in isolated single OH groups (between 463 K and 673 K, with a DTG peak at about 673 K), and a gradual decrease in single and geminal OH groups up to 1173 K [44]. The 2D zeolites with pillars of amorphous SiO_2_ show a very monotonous dihydroxylation, indicating a low content of vicinal silanols (observe that during synthesis, the samples were already subjected to calcination at 823 K to remove organic components, see Section 3); this inevitably led to the removal of part of the silanol groups as well. In addition, according to ^29^Si MAS NMR data [26], geminal silanols are absent. This allows us to conclude that isolated silanols are the main surface species in pillared zeolites.

Despite the fact that water release starts even below 313 K (initial temperature of the TG analysis) and the mass loss is not finished even above 1100 K and some hydroxyl groups still remain, a qualitative estimate for MOR-P provides that the ratio between water and hydroxyl protons is about 4:1. Of course, such an estimate is very approximate, but even so, it allows us to conclude that the developed inner surface of mesopores is covered with a significant number of hydroxyl groups.

In spite of the slightly higher *V*_tot_ value of MFI-P (see Table 1), MOR-P has essentially a higher water content. This suggests that water fills the mesoporous voids unevenly because water molecules are mainly located near the mesoporous surface, interacting with it, and the strength of this interaction is governed by the topology of the inner surface. It should be noted that the texture of both samples is quite similar, so the observed differences are presumably related to the interaction energy of water molecules with the elements of the geometric structure of the zeolite. The pillared MFI-P retains water more weakly—dehydration starts at a lower temperature, but is distributed in a wider region, which is associated with a greater heterogeneity of water adsorption energies as compared to mordenite.

### 2.3. ^1^H MAS NMR

**Figure 4:**
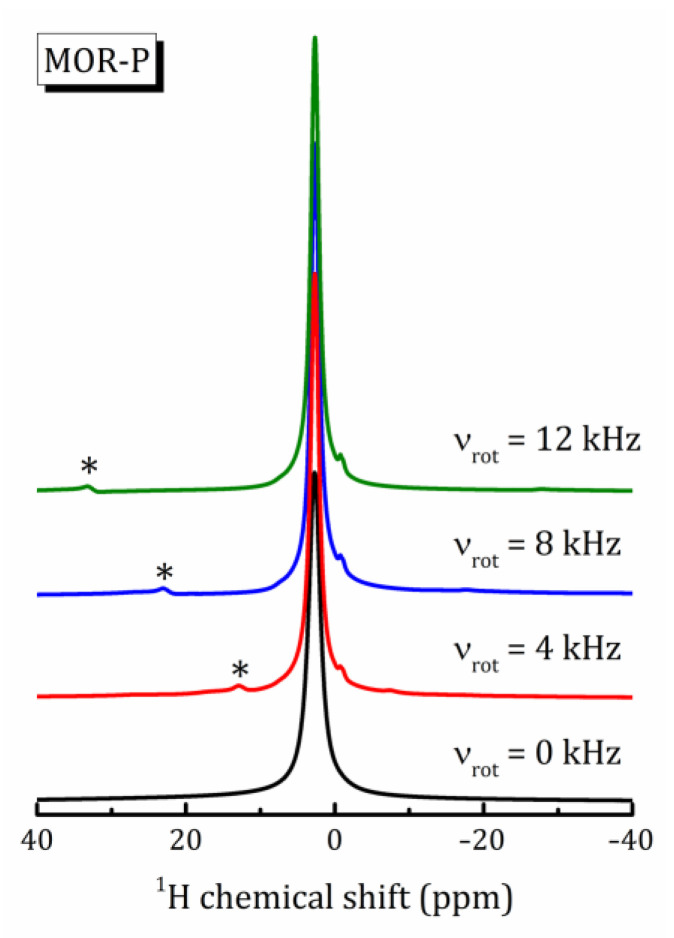
Static ^1^H NMR (ν_rot_ = 0) and MAS spectra recorded at rotor frequency ν_rot_ = 4, 8 and 12 kHz at T = 293 K in MOR-P; asterisks mark spinning sidebands.

^1^H NMR was applied to study the behavior of nanoconfined water molecules in pillared zeolites. Figure 4 shows the ^1^H spectra in MOR-P recorded at 293 K in static and spinning modes at various rotation frequencies ν_rot_. For the static mode, the signal represents a single peak at 2.7 ppm with a full width at half maximum (FWHM) Δν_1/2_ = 2 ppm. MAS results in improved spectral line resolution and a set of weak signals appears between −1 and 0 ppm. For MFI-P, the static ^1^H NMR spectrum represents a single peak at 3 ppm with Δν_1/2_ equal to 1.7 ppm. The spinning mode also leads to a slight narrowing of the line and reveals signals between −1 and 0 ppm.

**Figure 5:**
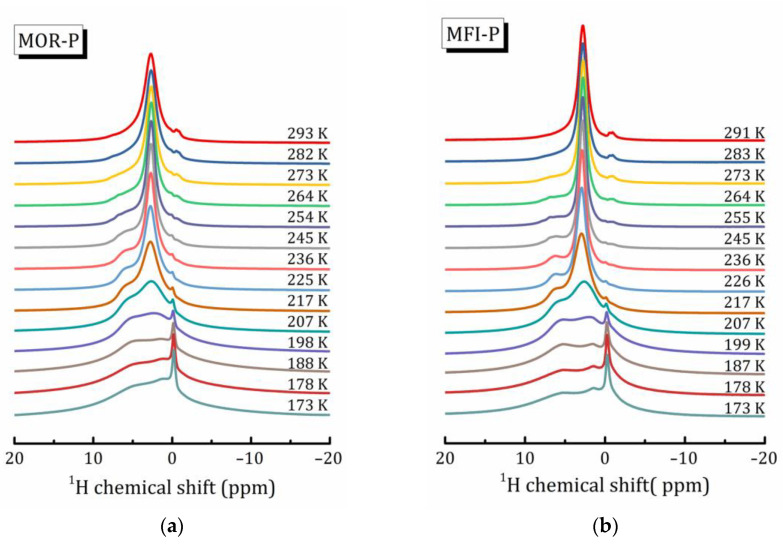
^1^H MAS NMR spectra (ν_rot_ = 12 kHz) recorded at various temperature for the MOR-P (**a**) and MFI-P (**b**) samples.

To study the behavior of nanoconfined water in more detail, ^1^H MAS NMR spectra were recorded at ν_rot_ = 12 kHz within the temperature range from 173 to 293 K. The temperature evolution of the spectra is shown in Figure 5. As can be seen, for both samples, the ^1^H signal shape changes in a similar way with the temperature decreasing: (i) declining and broadening of the signal at 2.7 ppm with the simultaneous appearance of a broad line near 5 ppm (let us denote these lines as L1 and L2, respectively); (ii) growing the intensity of the line at 0 ppm, whose linewidth Δν_1/2_ remains equal to 0.4 ppm even at 173 K.

Altogether, it indicates that upon cooling in both studied pillared zeolites with hierarchical porosity, adsorbed water undergoes structural transformations, which are affected by interactions with the inner pore surface.

**Figure 6:**
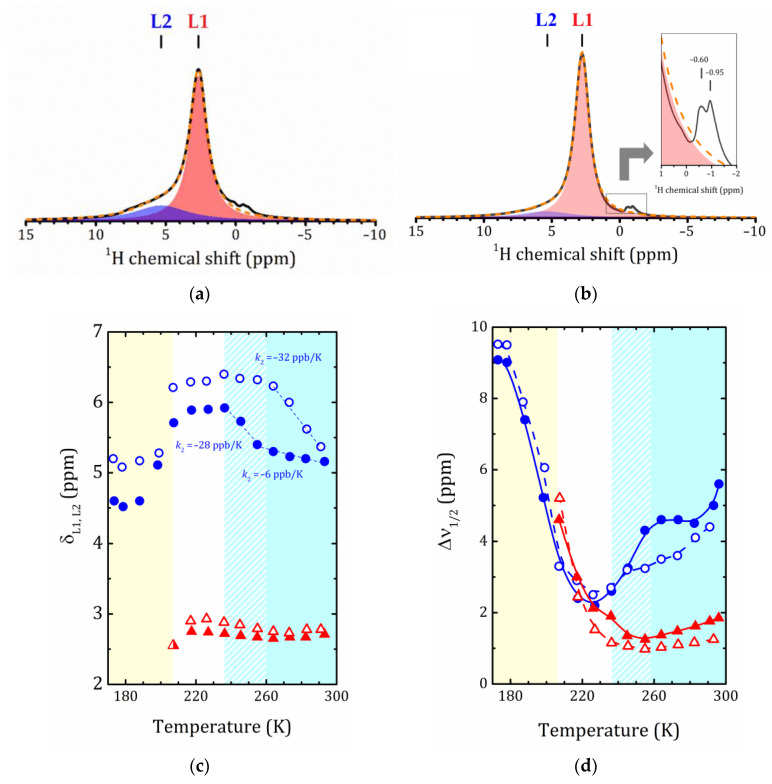
Decomposition of the ^1^H MAS NMR spectra into two Lorentz lines, L1 and L2, for MOR-P at 293 K (**a**) and MFI-P at 291 K (**b**); dashed lines show the sum fit; (**c**)—temperature dependence of the ^1^H isotropic chemical shift δ_L1_ and δ_L2_ of the L1 and L2 lines, respectively; dashed lines show the linear fitting by δ_L(*i*)_ = δ_0_ + *k*_(*i*)_*T*; the corresponding *k*_(*i*)_ values are provided in the plot; (**d**)—Δν_1/2_ of the L1 and L2 lines versus temperature in MOR-P and MFI-P; the lines are a guide to the eye. Triangles and circles correspond to L1 and L2, respectively; closed symbols—MOR-P; open symbols—MFI-P. The error bars are all smaller than data point symbol.

Let us now analyze the temperature dependencies of the spectral parameters of the individual lines. For both the MOR-P and MFI-P samples, the shape of the ^1^H MAS NMR signal near room temperature can be adequately described by the superposition of two Lorentzian lines: an intense L1 line at 2.7 ppm with a L2 shoulder near 5.2 ppm. Examples of such a decomposition are shown in Figure 6a,b. As shown in Figure 1, the L2 line can be attributed to protons of bulk water or silanols that have formed hydrogen bonds with water, or water protons bound to silanols. The unbound protons of water and free silanols have a chemical shift of about 1.5 ppm [8]. As discussed in Section 2.2, in the studied pillared zeolites, a significant amount of hydroxyls, mainly silanols, are present on the surface of the SiO_2_ pillars. This conclusion is consistent with ^29^Si NMR spectra [26]. It means that L1 represents an averaged line due to the fast exchange between water protons and surface –SiOH groups [8]. Meanwhile, L2 can be attributed to hydrogen-bonded water protons, which are not involved in the exchange. The weak signals between −1 and 0 ppm, shown in the inset in Figure 6b, can be attributed to non-framework Al species Al(OH)*_n_* (see Figure 1j), which appear in pillared zeolites after evacuation of CTAB upon calcination, as confirmed by ^27^Al MAS NMR [26].

Since the isotropic chemical shifts of protons reflect the electronic environment in which they are located in the system, any changes in the local electronic environment of the probed protons are accompanied by changes in the chemical shifts. Water molecules form an extensive network of hydrogen bonds with each other and with surface species, as shown in Figure 1. The strength of these bonds depends on the temperature and on the specific species involved (e.g., the bonding of the water proton to silanol oxygen is much stronger in silica than in bulk water [15].

The temperature dependence reflects the changes in hydrogen bond lengths (weakening of the strength due to bond elongation with increasing temperature). Hence, by tracking the temperature behavior of the isotropic chemical shift of protons, one can follow the changes in the hydrogen-bonding network of confined water, especially in a supercooling regime.

**Figure 7:**
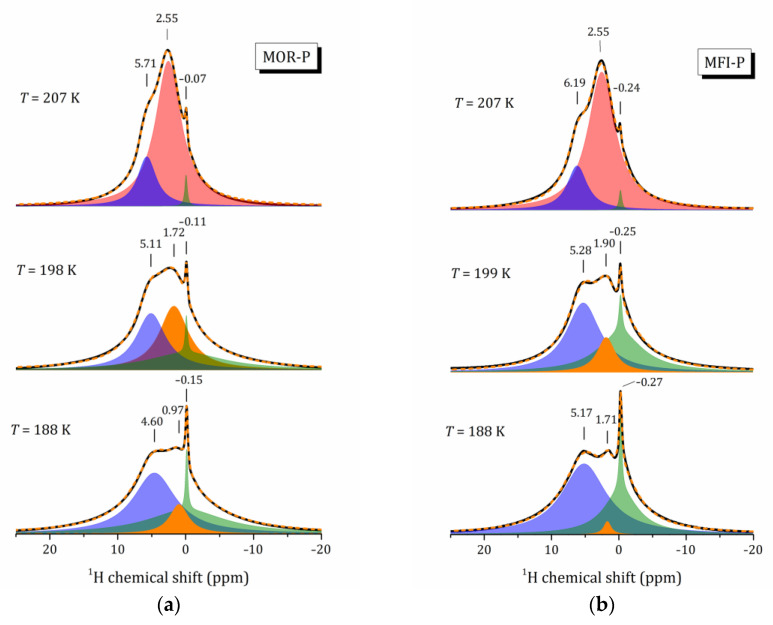
Decomposition of ^1^H MAS NMR spectra between 188 and 207 K for MOR-P (**a**) and MFI-P (**b**) samples.

Figure 6b shows the temperature dependences of the ^1^H isotropic chemical shift for the L1 and L2 lines, δ_L1_ and δ_L2_. All the dependencies exhibit complex behavior. For both studied samples, MOR-P and MFI-P, δ_L1_ decreases slightly with the decreasing temperature within the temperature range from 293 to 217 K, then increases (with a local minimum at 264 K) and disappears below 217 K (see Figure 7). δ_L2_ for MFI-P increases sharply upon cooling when temperature is varied from 291 to 264 K with a slope of –32 ± 1 ppb/K, then reaches a plateau of about 6.3 ppm. Between 217 and 199 K, δ_L2_ sharply decreases down to 5.2 ppm for MFI-P and then changes insignificantly upon further cooling. For MOR-P, the temperature dependence of δ_L2_ is rather similar to MFI-P, but the δ_L2_ values vary over a narrower range and have an inflection point at 255 K (with a slope of –6 ± 1 ppb/K above *T* = 255 K, and of –29 ± 4 ppb/K below it). Between 217 and 188 K, it decreases down to 4.6 ppm for MOR-P and remains almost constant thereafter. It is worth noting that as compared to MOR-P, MFI-P exhibits a systematically higher proton shifts that indicates stronger hydrogen bonding.

As can be seen from Figure 6c, the temperature behavior of the individual linewidths for both samples is rather similar: Δν_1/2_ of L1 initially remains practically unchanged upon cooling, and below 227 K it grows rapidly until it disappears. Such a temperature dependence of Δν_1/2_ is typical for atomic motion in solids. However, the linewidth Δν_1/2_ of L2 exhibits a very complex behavior (Figure 6c) that reflects the freezing out of this bulk water.

It should be noted that down to a temperature of 207 K, the relative intensities of these L1 and L2 lines remain practically unchanged. This means that till this temperature, the exchange process responsible for the appearance of the L1 line is still proceeding rapidly. The critical point between 199 and 207 K is the same for both zeolites, see the spectra in Figure 7: (i) the L1 line shifts, decreases in intensity and narrows; (ii) the line at about –0.1 ppm for MOR-P and –0.2 ppm for MFI-P, which can be represented as a superposition of a very narrow and a very broad components, begins to increase drastically; (iii) the intensity of the line at about 5 ppm that corresponds to hydrogen-bonded water protons and previously attributed only to water not interacting with the surface, starts to grow and broaden rapidly. Such a correlation in the change in line intensities can be due to the splitting of L1 because of the slowing down of the exchange process.

The presence of a narrow line below 200 K means that a part of protons is still very mobile. As reported in several studies [45,46,47], water located near the surface of mesopores and interacting with silanol groups on their surface exhibits rapid rotational motion even at low temperatures.

Now, let us discuss the temperature behavior of bulk water, shown as line L2 in Figure 6. Upon cooling, an increase in the chemical shift is observed, indicating a strengthening of hydrogen bonds (more noticeable for MFI-P that can be due to the somewhat larger mesopore size). As already noted, the fraction of bulk water in the range 293–207 K is practically unchanged and extends to about 20% of the total signal intensity, so presumably this line can be attributed to water in micropores. Let us remind ourselves that bulk water has four hydrogen bonds, which are quite strong. With a hydrophilic surface, water also forms hydrogen bonds, but only two. Moreover, unlike the hydrophilic surface of mesopores, which is completely covered by hydroxyl groups, the surface of micropores can be both hydrophilic and hydrophobic. In any case, above 207 K in the complex competition of enthalpic and entropic factors they are practically balanced.

A sharp increase in the linewidth of the L2 line at a temperature of about 178 K is associated with the freezing of bulk water. The motional narrowing of the NMR line takes place when the rate of fluctuation of the proton dipole–dipole interaction, which is generally described by a correlation time $\tau_{c}$, becomes of the order of magnitude of the rigid lattice. Generally, it is assumed that this thermally activated process proceeds according to the Arrhenius law:

(1) $$\tau_{c}=\tau_{0}exp\left(\frac{E_{a}}{k_{B}T}\right),$$

where $E_{a}$ is the activation energy; $k_{B}$ is the Boltzmann constant; and $\tau_{0}$ is a pre-exponential factor. The activation energy of the NMR line narrowing process $E_{a}$ can be estimated from the onset temperature $T_{0}$ using the Waugh–Fedin expression [48]:

(2) $$E_{a}(eV)=1.617\times 10^{-3}T_{0}(K).$$

For both pillared zeolite samples $T_{0}≅180$ K (see Figure 6c), that results in $E_{a}$ = 0.291 (eV) = 28.1 (kJ/mol). However, this estimation is very approximate. More information about the dynamic parameters of water motion can be accessed by proton NMR relaxation.

### 2.4. ^1^H Relaxation Study

**Figure 8:**
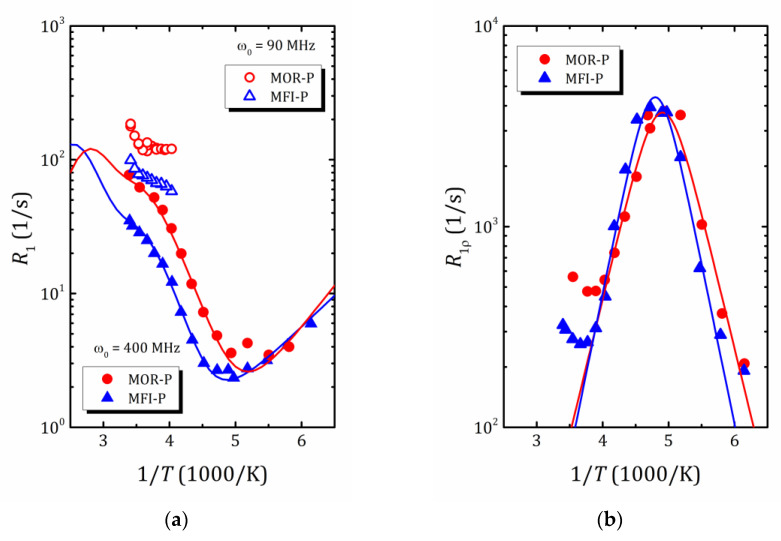
(**a**)—*R*_1_ relaxation rate at 400 MHz (solid symbols) and 90 MHz (open symbols) versus inverse temperature in MOR-P (triangles) and MFI-P (circles); solid lines show fit at 400 MHz using Equation (5); (**b**)—$R_{1\rho }$ relaxation rate at 400 MHz versus inverse temperature at locking pulse frequency ν_1_ = 40 kHz; solid lines represent fit using Equation (4). The error bars are all smaller than data point symbol.

Earlier, we reported the results of the ^1^H relaxation study of water mobility in a pillared mordenite sample [49]. The temperature dependence of the ^1^H spin-lattice relaxation rate *R*_1_ and the relaxation rate in the rotating frame *R*_1ρ_ indicated a complex behavior of nanoconfined water that can be considered within the framework of the modified exchange model [49,50,51]. To study the effect of texture on the mobility of confined water, the temperature dependences of the proton relaxation rates *R*_1_ and *R*_1ρ_ in MFI-P were measured. Over the whole temperature range investigated, the ^1^H magnetization recovery curves exhibit a mono-exponential behavior. The experimental *R*_1_(1/*T*) and *R*_1ρ_(1/T) data for MFI-P are plotted in Figure 8a,b, respectively. The data for MOR-P from Ref. [49] are provided for a comparison. As can be seen, at 400 MHz *R*_1_ for MFI-P exhibits a complex temperature behavior similar to that observed for MOR-P, but with a systematically shorter spin-lattice relaxation time.

The main issues responsible for the NMR relaxation in the studied systems are fluctuating strengths of proton–proton dipole coupling. For liquids, the spin lattice relaxation is usually well described within the Bloembergen–Purcell–Pound (BPP) model [52]:

(3) $$R_{1}=G(0)\cdot \left[\frac{1}{3}\cdot \frac{{2\tau }_{c}}{1+{\left(\omega_{0}\tau_{c}\right)}^{2}}+\frac{4}{3}\cdot \frac{{2\tau }_{c}}{1+{\left(2\omega_{0}\tau_{c}\right)}^{2}}\right],$$

Here, $\omega_{0}^{}$ is the nuclear resonance frequency; G(0) contains information on the mutual arrangement of nuclei; the correlation time $\tau_{c}$ obeys the Arrhenius law in Equation (2). This model suggests a Λ-shape of *R*_1_(1/*T*) dependence with a maximum at $\omega_{0}\tau_{c}\approx 1$ [22]. For slow spin motion, which is normally the case of solids, the maximum is shifted towards the high temperature and often lies beyond the accessible temperature range. That makes it difficult to determine the motion parameters {$E_{a}$, $\tau_{0}$}. To study slow spin motion, a sip-locking technique can be applied. The application of the locking pulse $\omega_{1}$ allows the maximum to shift into the accessible temperature window, and the temperature dependence of the spin-lattice relaxation rate *R*_1ρ_, that describes the evolution of the spin system in this rf field, can be written as follows [22]:

(4) $$R_{1\rho }=G\left(0\right)\cdot \left[\frac{1}{2}j\left(\omega_{1}\right)+\frac{5}{6}j\left(\omega_{0}\right)+\frac{1}{3}j\left(2\omega_{0}\right)\right].$$

As can be seen from Figure 8b, for both samples, the $R_{1\rho }(1/T)$ dependence exhibits a very pronounced maximum in the middle of the temperature range. The approximation using Equation (4), shown in Figure 8b by solid lines, provides very close values of activation energy, 28.9 and 30.7 kJ/mol for MOR-P and MFI-P samples, which is very close to the value estimated from the Waugh–Fedin expression.

It should be noted that although this maximum is not observed in Figure 8a, this type of motion contributes to the total $R_{1}(1/T)$ and must be taken into account when fitting the experimental data. Moreover, the spin-lattice relaxation times measured at 90 MHz also clearly indicate the beginning of the formation of a new maximum in the high temperature range. Following the exchange model developed for the proton spin-lattice relaxation of water molecules confined in the pillared mordenite [49], the temperature dependence of ^1^H $T_{1}$ in MFI-P was fitted by the following equation:

(5) $$R_{1}=G\left(0\right)\cdot \left\{p_{A}\left(T\right)\;\cdot \left[\frac{1}{3}j_{A}\left(\omega_{0}\right)+\frac{4}{3}j_{A}\left(2\omega_{0}\right)\right]+p_{B}\left(T\right)\cdot \left[\frac{1}{3}j_{B}\left(\omega_{0}\right)+\frac{4}{3}j_{B}\left(2\omega_{0}\right)\right]+p_{C}\cdot \left[\frac{1}{3}j_{C}\left(\omega_{0}\right)+\frac{4}{3}j_{C}\left(2\omega_{0}\right)\right]\right\},$$

Here, $p_{A}\left(T\right)+p_{B}\left(T\right)+p_{C}=1$ are relative fractions of water protons participating in different types of motion: $p_{A}\left(T\right)$ is a fraction of water interacting with the zeolite surface but having limited mobility; $p_{B}\left(T\right)$ is a fraction of water interacting with the zeolite surface but having fast rotational motion, $p_{C}$ is a fraction of water molecules participating in translational motion. Within the framework of this model, it is assumed that the ratio between fractions A and B is temperature-dependent and can be described by the following expressions [51]:

(6) $$p_{A}\left(T\right)=\frac{\left(1-p_{C}\right)}{2}\cdot \left[1+\mathrm{erf}\left(\frac{T-\Theta_{1}}{2\Theta_{2}}\right)\right];$$

(7) $$p_{B}\left(T\right)=1-p_{A}\left(T\right)-p_{C},$$

where erf(*x*) is the error function with parameters $\Theta_{1}$ (median) and $\Theta_{2}$ (slope control).

The results of the approximation obtained within this model for $\omega_{0}=400$ MHz are shown in Figure 8a by solid lines. The parameters of the model are listed in Table 2.

## 3. Discussion

First of all, let us summarize the three kinds of surfaces that are clearly present in our MOR-P and MFI-P materials, on which water can be adsorbed. First, they are segments of zeolite channels that cross the 2D zeolite layers; second, they are the surface of crystalline aluminosilicate 2D zeolite layers exposed in the spatial 2D gap between the zeolite layers; and finally, they are the surface of amorphous silica that make up the pillars that separate the zeolite layers and keep them from collapse. In addition, we note that the slit-shaped pores are of sufficient thickness, as well as extent in the other two dimensions, so that the states of water molecules in contact with the surface and in the pore volume can, at least theoretically, differ. This entire system of hierarchical pores in both zeolites can adsorb, and indeed does adsorb water, although despite the apparent similarity of the geometry of the slit-shaped pores in MOR-P and MFI-P their properties are quite different.

In this confined space, which has a mosaic pattern of different types of surfaces, water molecules are involved in translational and rotational motion, which freezes at a temperature of about 180 K that is characteristic of nanoconfined water. But even at such a low temperature, some of the protons remain very mobile, as evidenced by the presence of a narrow ^1^H NMR line (see Figure 7). The latter seems to be related to the decrease in the filling factor of pores by water molecules and the restructuring of hydrogen bounding network due to nanoconfinement [53]. All these processes are characterized by certain activation energies.

The highest activation energy $E_{a}^{A}$, determined from the $R_{1}(1/T)$ dependence, is of about 30 kJ/mol and is very close to the *E*_a_ value found from the spin-locking experiment and from the motional narrowing of the ^1^H NMR line (see Table 2). Altogether, this allows us to attribute this energy to the freezing of nanoconfined water. The lowest activation energy $E_{a}^{B}$ (12 kJ/mol for pillared mordenite and 9 kJ/mol for pillared MFI) and the shortest correlation time can be related to very fast rotational motion and agree well with the results of MD simulation of water behavior in MCM-41 [54].

Finally, the value of $E_{a}^{C}$ (23.6 and 26.0 kJ/mol for MOR-P and MFI-P, respectively), which determines the slope of the 1/*T*_1_(1/*T*) dependence within the temperature range 230 < T < 300, is very close to the activation energy of intracrystalline diffusion in microporous zeolites probed by static field gradient NMR (mordenite) [41] and quasi-elastic neutron scattering (zeolites NaX, NaY) [55] and is associated with the translational motion of confined water molecules. It can be assumed that the translational movement of water molecules is determined mainly by the micropores of the zeolite. Nevertheless, to confirm this assumption, both additional measurements of the diffusion of water molecules and experiments with larger molecules, the movement of which through the micropores of zeolite is difficult due to sieve effects, are necessary. However, such studies are beyond the scope of this work; once completed, the results will be published elsewhere.

To compare the influence of pore size and connectivity on the parameters of water motion, we listed in Table 3 the activation energies of rotational and translational motion of both bulk water and water in different zeolites (both micro- and mesoporous) and in mesoporous silicas.

It is interesting to note that the reorientation motion of water in pillared zeolites has lower activation energies than in bulk water (experimental data [56,57]), and mesoporous MCM-41 (estimates from molecular dynamics [54]), although close to the calculated values in microporous NaA zeolite [58].

For translational motion, the scatter in the activation energy is even larger. It is known that for supercooled water, the activation energy of translational motion depends on temperature and does not obey the Arrhenius law [62]. Nevertheless, when interpreting experimental data, the Arrhenius dependence is often used in a limited temperature range. The translational motion of water in mesoporous silicas is characterized by a rather high activation energy, whereas in microporous zeolites its value is close to the values for bulk water (it should be noted that this parameter is very sensitive to the Si/Al ratio and to water loading: it increases with Si/Al growth [55,63]), and decreases with water loading [55]. The values of the activation energies of translational motion for pillared zeolites of 23.6 and 26.0 kJ/mol obtained in this work are very close to those for microporous zeolites and free water. Moreover, for mesoporous NaA zeolite, in which mesopores were obtained by direct synthesis in the presence of quaternary ammonium cationic organosilane surfactant as a mesopore-generating agent, the activation energy of self-diffusion is very high [61]. This allows us to suppose that the ordering of micropores that takes place in pillared zeolites synthetized in the presence of OSDA [37], and their mutual arrangement, could be responsible for such a low activation energy in the zeolites with hierarchical porosity studied in the present work. In other words, the translational motion of water (more precisely, its part) is influenced by the orderliness of the mutual arrangement of micro- and mesopores.

## 4. Materials and Methods

Synthesis of samples was carried out according to the procedure described in detail in our previous works [26,37]. Pillaring of the obtained materials was performed as proposed by Na et al. [64]. The preparation process involved four steps: (i) preparation of lamellar zeolites by self-assembling method using cetyltrimethylammonium bromide (CTAB) and polyethylene glycol (PEG) as agents creating mesopores in a one-pot synthesis (tetrapropylammonium bromide (TPABr) was additionally used for ZSM-5 synthesis); (ii) introduction of tetraethoxysilane (TEOS) molecules into the interlamellar space occupied by CTAB layer; (iii) TEOS hydrolysis in water, to form amorphous SiO_2_; (iv) calcination, to remove organic molecules, at 823 K. More information on the details of each step are provided in Ref. [26]. The obtained samples are designated as MOR-P and MFI-P.

Thermogravimetric (TG) analysis was performed using a Netzsch TG 209 F1 Libra instrument in the temperature range from 313 to 1173 K, at a heating rate of 10 K/min, in an argon flow at a rate of 30 mL/min.

The texture of the samples was studied by N_2_ porosimetry. Nitrogen physisorption isotherms (both adsorption and desorption branches) were recorded at 77 K on a QuadraSorb SI instrument. Prior to analysis, the studied samples were outgassed under vacuum for 6 h at a temperature of 573 K. The data analysis was performed with a QuadraWin (Version 5.11) software. Apparent surface areas were determined by the Brunauer–Emmett–Teller (BET) method, pore size distributions were obtained by nonlocal density functional theory (NLDFT), and mesopore surface areas were evaluated by *t*-plots analysis.

^1^H NMR experiments were carried out using a Bruker Avance IIITM 400 MHz solid-state NMR spectrometer (operating with Topspin version 3.2) using a double-resonance 4 mm low temperature magic angle spinning (MAS) probe. Tetramethylsilane (TMS) was used as an external standard. The temperature was varied within a temperature range from 173 to 293 K and controlled to an accuracy of 0.5 K. In the MAS experiment, the rotation frequency was equal to 12 kHz. The spin-lattice relaxation times *T*_1_ were measured using the so-called inversion-recovery method (180°–τ–90°).

To measure spin-lattice relaxation times in a rotating frame *T*_1ρ_, the spin-locking technique was applied. In our experiment, the *B*_1_ field amplitude was chosen at about 1mT, corresponding to a *B*_1_ frequency of 40 kHz. *T*_1_ relaxation times were also measured at 90 MHz using a Bruker SXP 4-100 spectrometer in a temperature range from 248 to 273 K with an accuracy of 0.5 K.

## 5. Conclusions

A comprehensive analysis of the behavior of water molecules, nanoconfined in mordenite and ZSM-5 zeolites, pillared with amorphous SiO_2_, was carried out applying ^1^H NMR spectroscopy and relaxation within the temperature range from 173 to 293 K. The pore distribution in these pillared zeolites was characterized by N_2_ porosimetry; the water release process was studied by TGA. The main conclusions can be summarized as follows.

Both pillared zeolites are characterized by a similar texture but slightly differ in the pore size distribution. MOR-P shows a more uniform distribution of mesopores and a slightly lower average pore size.

The TG/DTG analysis suggests that water molecules in both zeolites are mainly located near the mesoporous surface, interacting with it through hydroxyls which are present in a large amount. The strength of this interaction is determined by the inner surface topology. Compared to MOR-P, MFI-P retains water more weakly, exhibiting a large heterogeneity of water adsorption energies.

Temperature evolution of ^1^H MAS NMR spectra evidences that water in such a nanoconfinement with hierarchically organized voids (micropores in 2D lamellae of zeolites and mesopores of about 4 nm in size between zeolite lamellae) experiences complex temperature behavior.

The temperature dependence of proton spin-lattice relaxation suggests the presence of three different processes with characteristic correlation times (activation energies): freezing, fast rotation, and translational motion of water. The water freezing for both pillared zeolites occurs near 180 K that corresponds to the activation energy of about 30 kJ/mol. The activation energy of water rotational motion (12 and 9 kJ/mol for MOR-P and MFI-P, respectively) is lower than that for bulk water that can be related with a low water density in mesopores.

The activation energy of translational motion for MOR-P and MFI-P zeolites, 23.6 and 26.0 kJ/mol, respectively, is very close to the values in microporous zeolites, but noticeably lower than for water in mesoporous silicas or zeolites with a similar mesopore size, but with disordered mesoporosity. This suggests that in pillared zeolites, as in silicas, only a part of the water interacts with silanol groups on the surface of the SiO_2_ pillars that form mesopores, and this part of the water fraction is characterized by fast rotational but slow translational motion; another part of water (25 and 15% for pillared mordenite and ZSM-5, respectively) participates in translational motion through the ordered micropores of zeolite. This indirectly indicates that the diffusion process in zeolites with hierarchical porosity is influenced not only by the presence of mesopores, but also by the mutual arrangement of meso- and micropores, and the translational motion of water molecules is determined mainly by the zeolite micropores.

## Figures and Tables

**Table 1:** Results of *t*-plot and BET methods, as well as results of SAXS, DFT, and BJH.

Properties	MOR-P	MFI-P
*S*_meso+ext_ (m^2^/g)	610	580
*S*_ext_ (m^2^/g)	125	125
***S*_meso_ (m^2^/g)**	**475**	**455**
***S*_BET_ (m^2^/g)**	**679**	**658**
*S_BJH_* (m^2^/g)	*247*	*283*
*S_DFT_* (m^2^/g)	*790*	*800*
*S*_micro_ (m^2^/g)	69	78
*V_tot_* (cm^3^/g)	*0.44*	*0.47*
*V*_micro_ (cm^3^/g)	0.037	0.043
***d*_0_ (SAXS) (nm)**	**3.3** ^1^	**3.2** ^1^
***d* (DFT) (nm)**	**4.0**	**4.4**
*d (BJH)* (nm)	*3.6*	*3.8*

^1^ Void interlamellar space, calculated using data from Refs. [26,37].

**Table 2:** Parameters of hydrogen motion in the MOR-P and MFI-P samples, as determined from the temperature dependences of the spin-lattice relaxation times *T*_1_ and *T*_1ρ_ (*E*_a_ and *τ*_0_), as well as *E*_a_ estimated from motional narrowing of the linewidth. Parameters for MOR-P determined from *T*_1_ and *T*_1ρ_ temperature dependence are taken from Ref. [49].

Parameters	MOR-P	MFI-P
	From *R*_1_ Experiment (Exchange Model)	
$E_{a}^{A}$ (kJ/mol)	29 ± 1	30 ± 1
$\tau_{0}^{A}$ (s)	1.5 ± 0.5 × 10^−14^	3.0 ± 0.5 × 10^−14^
$E_{a}^{B}$ (kJ/mol)	12 ± 1	9 ± 1
$\tau_{0}^{B}$ (s)	8.0 ± 0.5 × 10^−16^	6.5 ± 0.5 × 10^−16^
$p_{A}^{}$	Equation (6)	Equation (6)
$p_{B}^{}$	Equation (7)	Equation (7)
$E_{a}^{C}$ (kJ/mol)	23.6 ± 0.5	26.0 ± 0.5
$\tau_{0}^{C}$ (s)	10 ± 1 × 10^−15^	4.5 ± 0.5 × 10^−15^
$p_{C}^{}$	0.25	0.15
$\Theta_{1}$ (K)	210 ± 10	220 ± 10
$\Theta_{2}$ (K)	15 ± 5	10 ± 5
	**From *R*_1ρ_ experiment**	
*E*_a_ (kJ/mol)	28.9 ± 0.2	30.7 ± 0.5
*τ*_0_ (s)	1.5 ± 0.1 × 10^−13^	8.1 ± 0.1 × 10^−14^
$\tau_{c(298K)}^{}$ (s)	1.7 × 10^−8^	1.9 × 10^−8^
	**From Waugh–Fedin expression (Equation (2))**	
*E*_a_ (kJ/mol)	28.1 ± 0.4	28.1 ± 0.4

**Table 3:** Activation energy for rotation and translation motion of bulk and nanoconfined water in zeolites and silicas as determined by different methods: NMR spin-lattice relaxation (NMR, *T*_1_); spin-spin relaxation (NMR, *T*_2_); self-diffusion (NMR, *D*); Kerr effect; molecular dynamics (MD); quasielastic neutron scattering (QENS).

Probe	Micro/Meso- Pore Size (nm)	Temperature Range (K)	Method	*E*_a_ (kJ/mol)	Ref.
**Rotation motion**					
Bulk water	−	273–298	NMR, *T*_1_	13.8	[56]
Bulk water	−	308–353	NMR, *T*_1_	19.2	[56]
Bulk water	−	275−365	Kerr effect	15.6	[57]
MCM-41	−/2.4	200−300	MD	18 ± 1	[54]
Pillared mordenite	0.7/4.0	173−293	NMR, *T*_1_	12 ± 1	[49]
Pillared ZSM-5	0.55/4.4	173−291	NMR, *T*_1_	9 ± 1	This work
NaA	0.41/−	278−353	MD	7.5 ±1.5	[58]
**Translation motion**					
Bulk water	−	274–288	NMR, *D*	19.7	[59]
Bulk water	−	288–318	NMR, *D*	17.6	[59]
MCM-41	−/2.4	200−300	MD	35 ± 1	[54]
MCM-41	−/2.4	180–220	NMR, *T*_2_	37.2 0.9	[54]
MCM-41	−/2.1	207–270	NMR, *D*	38.6	[60]
Mesoporous NaA	0.41/5.0	250–310	NMR, *D*	56.2	[61]
Pillared mordenite	0.7/4.0	173−293	NMR, *T*_1_	23.6 ± 0.5	[49]
Pillared ZSM-5	0.55/4.4	173−291	NMR, *T*_1_	26.0 ± 0.5	This work
Na-mordenite	0.7/−	230–300	NMR, *D*	25.6 ± 0.5	[41]
NaX	0.74/−	300	QENS	19 ± 1	[55]
NaY	0.74/−	300	QENS	16.5	[55]

## Data Availability

The data that support the findings of this study are available from the corresponding author, (M.G.S), upon reasonable request.
